# Malignant Pilomatricoma of the Lower Extremity: A Difficult and Rare Diagnosis

**DOI:** 10.7759/cureus.21957

**Published:** 2022-02-06

**Authors:** Chaitra Subramanyam, Paige Dyrek, Xu Yao, Martin H Kay

**Affiliations:** 1 Dermatology, Western University of Health Sciences, Lebanon, USA; 2 Orthopedics, Stanford University, Redwood City, USA; 3 Pathology, Providence Saint Joseph Medical Center, Burbank, USA; 4 Dermatology, Keck School of Medicine, University of Southern California, Los Angeles, USA

**Keywords:** pilomatrix carcinoma, cutaneous lesions, dermatology oncology, cutaneous oncology, malignant pilomatricoma

## Abstract

Malignant pilomatricoma is a rare cutaneous malignancy that is commonly found on the head and neck. We present a patient with malignant pilomatricoma of the lower extremity with intralesional calcification and giant cells, confirmed by histopathology. This patient’s case represents a clinically important variation of malignant pilomatricoma in an uncommon anatomical location.

## Introduction

Malignant pilomatricoma is a rare cutaneous malignancy that is typically found on the head and neck. Also known as pilomatrix carcinoma, these are aggressive tumors that have a strong tendency to recur and metastasize [1,2]. Herein, we report and discuss a patient with malignant pilomatricoma of the lower extremity with histopathological intralesional calcification and giant cells.

## Case presentation

**Figure 1:**
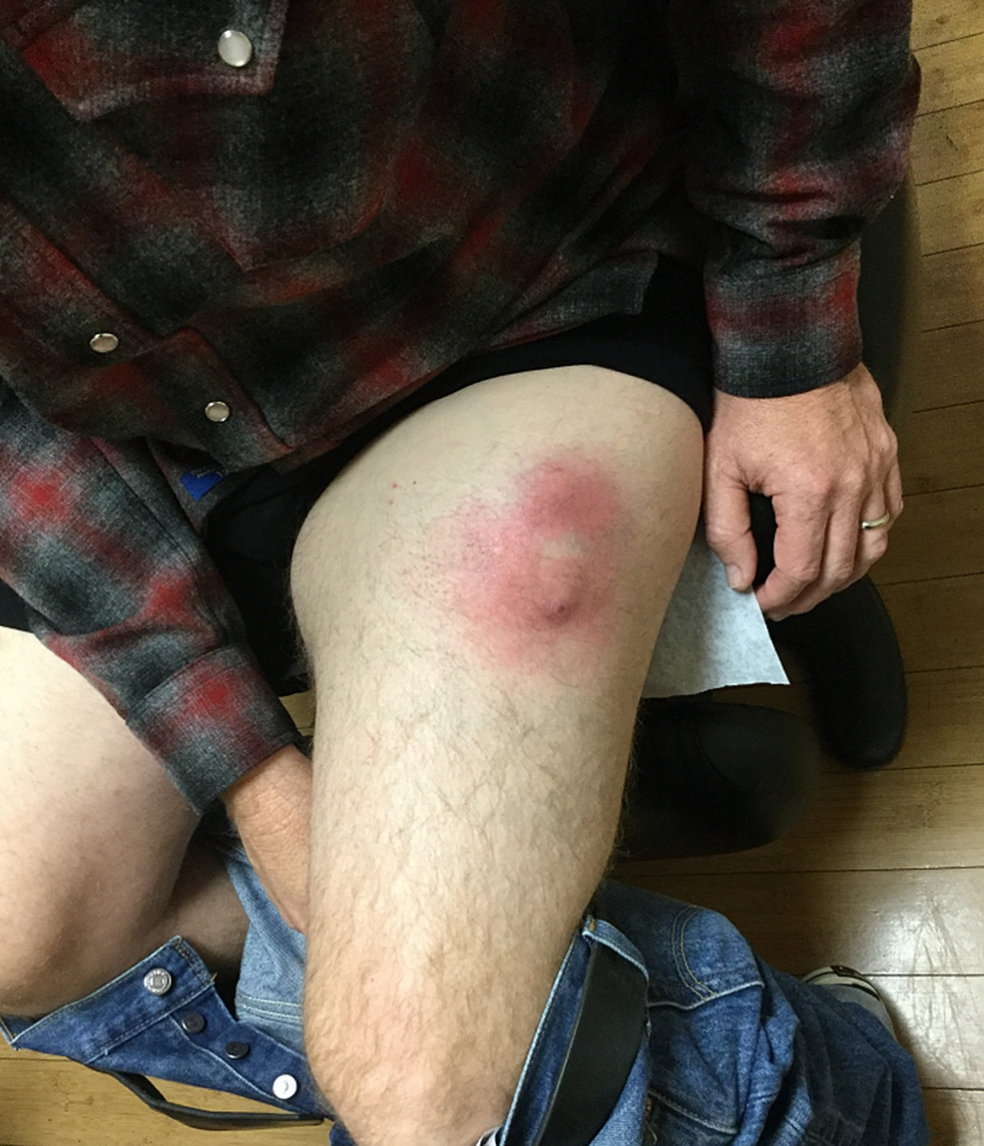
Anterior view of the left leg showing erythematous subcutaneous nodule with surrounding erythema

A 51-year-old Caucasian male with no history of skin cancer presented with a six-month history of a slow-growing mass on his left thigh. Physical examination showed a 6cm x 6cm erythematous subcutaneous nodule on the lateral aspect of his left proximal thigh with surrounding inflammation (Figure 1). The lesion was initially suspected to be an infected cyst and the patient was prescribed a course of oral antibiotics and advised to apply warm compresses. 

**Figure 2:**
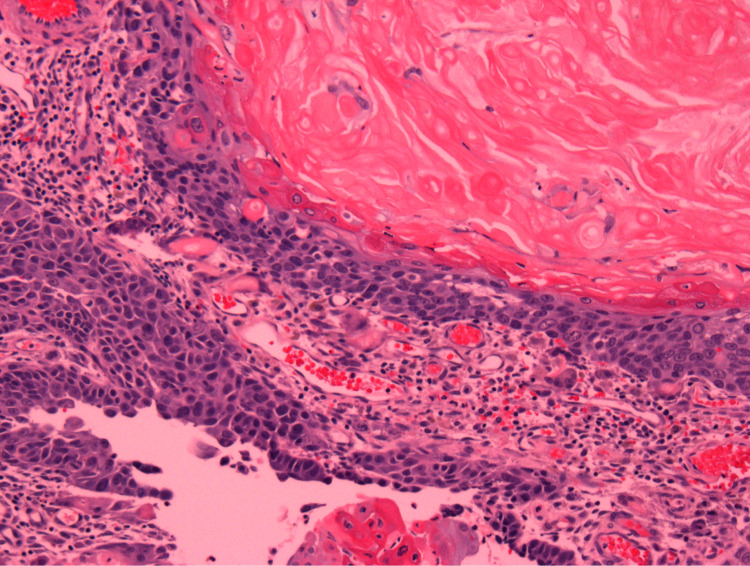
Intermediate power view (100x) showing basaloid tumor cells with "ghost cells" and keratinization

He returned one week later without significant improvement, at that time an excisional biopsy was performed and pathology showed calcified homogenous keratin material with ghost cells, separate multinucleated giant cells, and lymphocytes, consistent with pilomatricoma (Figure 2). Initial conservative excision of a presumed benign pilomatricoma did not remove all of the tumors due to extensive calcification found and the patient was referred for further surgical management. 

**Figure 3:**
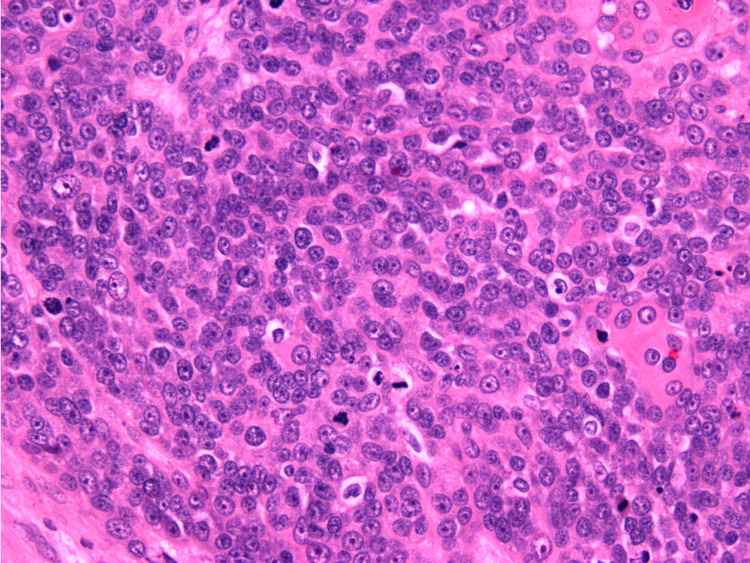
High power view (400x) showing basaloid tumor with frequent mitosis and karyorrhexis

**Figure 4:**
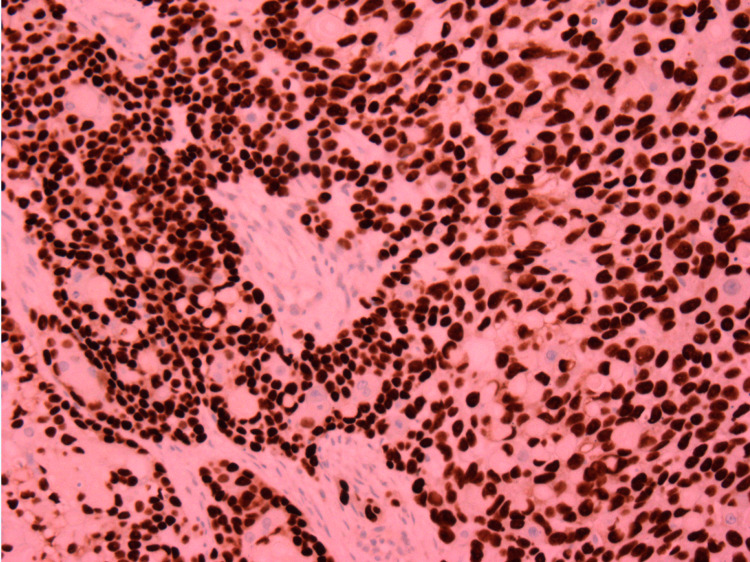
Tumor cells are strongly positive for p16 by immunohistochemical stain

Prior to re-excision, an MRI of the left upper leg was performed which was significant for a lobulated soft tissue mass. The mass appearance was concerning for a primary soft tissue sarcoma or metastatic disease of lymphoma type. Partial excision of the tumor returned a diagnosis of invasive carcinoma, morphologically most consistent with a malignant pilomatricoma. Pathological findings included regular tumor lobules composed of basaloid cells with atypia, numerous mitosis apoptotic figures embedded in a desmoplastic stroma, and centrally located “shadow” or “ghost” cells (Figure 3). Immunohistochemistry was positive for keratin, p63, p16, and beta-catenin (Figure 4). Radical excision with split-thickness grafting was performed and margins of resection were negative with no lymphovascular (LVI) or perineural invasion (PNI). 

Adjuvant radiotherapy was discussed but not recommended as the tumor was excised with adequate surgical margins and did not display high-risk features (LVI or PNI). The patient agreed to radiologic surveillance every three months for the first two years with alternating MRI and positron emission tomography-computed tomography (PET-CT) scans and dermatological follow-up every three months.

## Discussion

Malignant pilomatricoma is a rare, locally aggressive neoplasm originating from follicular matrix cells [1]. Although only about 130 cases have been documented in English literature, malignant pilomatricomas are clinically important as they demonstrate locally aggressive behavior with a strong tendency to recur and metastasize [1,2]. Also known as pilomatrix carcinomas, most malignant pilomatricomas arise on the head and neck region, predominantly affect Caucasian males, and have a bimodal distribution in the sixth and seventh decades and the first three decades [3,4]. Only 11% of cases have been documented to occur on the lower extremity, as in this patient [3]. Typical presentation includes a solitary, slow-growing, and firm subcutaneous lesion that is often misdiagnosed for its benign equivalent due to similar histologic findings [5]. Both benign and malignant pilomatricomas exhibit nests of basaloid cells and eosinophilic, enucleated ghost cells. The additional presence of nuclear pleomorphism, cellular atypia, numerous mitoses, and areas of necrosis with contiguous desmoplasia can help differentiate the tumors [1-3,5]. Interestingly, intralesional calcification and giant cells occur rarely in these tumors, with a review of 35 different malignant pilomatricoma lesions revealing four and one cases with these findings, respectively [4]. The microscopic minutiae are especially important because there are no specific histologic criteria nor differentiating immunohistochemical markers for diagnosing malignant pilomatricoma. Following diagnosis, wide local excision is preferred as it results in a local recurrence rate of 23% as compared to 83% with simple excision [2]. Increased rate of recurrence is associated with higher rate of metastasis with the most common sites being regional lymph nodes and lungs [1,2]. Although systemic chemotherapy has not proven to be beneficial, adjunctive radiotherapy may be considered in cases of recurrence or metastasis [3]. Given the importance of correct diagnosis to surveil for recurrence and metastasis, we encourage continued work to develop high-quality evidence in the pathogenesis and treatment of malignant pilomatricoma.

## Conclusions

Malignant pilomatricoma is a rare cutaneous lesion that can present on the lower extremities with intralesional calcification and multinucleated giant cells. Wide local excision can be curative for this carcinoma. Adjunctive radiotherapy can be considered in cases of recurrence or metastasis, where the most common locations for metastasis are regional lymph nodes and the lungs.
